# Conspecific coprophagy stimulates normal development in a germ-free model invertebrate

**DOI:** 10.7717/peerj.6914

**Published:** 2019-05-13

**Authors:** Benjamin C. Jahnes, Madeline Herrmann, Zakee L. Sabree

**Affiliations:** 1 Department of Microbiology, The Ohio State University, Columbus, OH, USA; 2 Department of Evolution, Ecology and Organismal Biology, The Ohio State University, Columbus, OH, USA

**Keywords:** Germ-free insects, Gut microbiome, Development

## Abstract

Microbial assemblages residing within and on animal gastric tissues contribute to various host beneficial processes that include diet accessibility and nutrient provisioning, and we sought to examine the degree to which intergenerational and community-acquired gut bacteria impact development in a tractable germ-free (GF) invertebrate model system. Coprophagy is a common behavior in cockroaches and termites that provides access to both nutrients and the primary means by which juveniles are inoculated with beneficial gut bacteria. This hypothesis was tested in the American cockroach (*Periplaneta americana*) by interfering with this means of acquiring gut bacteria, which resulted in GF insects that exhibited prolonged growth rates and gut tissue dysmorphias relative to wild-type (WT) *P. americana*. Conventionalization of GF *P. americana* via consumption of frass (feces) from conspecifics and siblings reared under non-sterile conditions resulted in colonization of *P. americana* gut tissues by a diverse microbial community and a significant (*p* < 0.05) recovery of WT level growth and hindgut tissue development phenotypes. These data suggest that coprophagy is essential for normal gut tissue and organismal development by introducing beneficial gut bacteria to *P. americana*, and that the GF *P. americana* model system is a useful system for examining how gut bacteria impact host outcomes.

## Introduction

Host-associated bacteria are increasingly recognized as being inextricably involved in the health, development and evolution of their animal hosts, and the digestive tract is a major intersection for the host and its environment, and its gut bacterial consortia. In vertebrates, microbial ecosystems located within animal digestive tracts, hereafter referred to as “gut microbiota,” (Yasuda et al., 2015; Costea et al., 2018) can impact host health, behavior, dietary nutrient accessibility, infectious disease susceptibility, and disease morbidity (Sonnenburg & Bäckhed, 2016; Koppel, Maini Rekdal & Balskus, 2017; Parker et al., 2017; Foster, Rinaman & Cryan, 2017; Zhao & Elson, 2018). Invertebrates also harbor sparse-to-abundant multispecies microbial communities within gastric tissues that can be as specialized as those observed in vertebrates (Dillon & Dillon, 2004; Engel & Moran, 2013; Douglas, 2015), and the global distribution, trophic diversity, ecological contributions, and agricultural, environmental and human health impacts of insects have stimulated interest in insect-gut microbiota interactions. Although host diet accessibility and nutrient metabolism has been a major focus of insect host-gut microbiota work (Six, 2013; Engel & Moran, 2013; Brune & Dietrich, 2015; Peterson & Scharf, 2016; Kwong & Moran, 2016; Bonilla-Rosso & Engel, 2018), these microbial assemblages have also been shown to participate in zoonotic infections (Aksoy, 2018), mediate plant-insect interactions (Hansen & Moran, 2014; Casteel & Hansen, 2014; Shikano et al., 2017), shape host behavior (Keesey et al., 2017), and stimulate immune system functions (Ryu et al., 2008; Buchon, Broderick & Lemaitre, 2013) and host growth and development (Coon et al., 2014; Zheng et al., 2017). When the gut microbiota is comprised of, or dominated by, a single or few taxa, it is possible to link individual taxa to host benefits, as observed in *Riptortus pedestris* stink bugs (*Burkholderia* that confer pesticide resistance, (Kikuchi, Hosokawa & Fukatsu, 2007; Itoh et al., 2018)) and in honey bees (*Gilliamella* that degrades pollen constituents and *Bifidobacterium asteroids* that produce growth-promoting hormones, (Engel, Martinson & Moran, 2012; Kešnerová et al., 2017). Additionally, amenability of these insect models, as well as *Drosophila* fruit flies (Apidianakis & Rahme, 2011), to axenic/germ-free (GF) rearing has greatly facilitated efforts to assign discrete functions to specific gut taxa.

Among invertebrate model systems, cockroaches are also proving to be useful for exploring host-gut microbiota interactions. The gut microbiota of omnivorous cockroaches had been characterized primarily by cultivation and microscopy approaches (Bracke, Cruden & Markovetz, 1979; Cruden & Markovetz, 1980, 1987; Umunnabuike & Irokanulo, 1986; Dillon & Dillon, 2004; Thompson et al., 2012) until cultivation-independent community profiling (i.e., 16S rRNA gene amplicon sequencing) approaches became widely available. Tremendous phylogenetic diversity, spanning over 20 bacterial phyla (Mikaelyan et al., 2015), has been detected in cockroach guts, with members of the Bacteroidetes, Firmicutes, Fusobacteria, and Proteobacteria comprising the majority of the gut microbiota, and diet appears to exert a significant impact on presence and relative abundance of the gut microbiota (Schauer, Thompson & Brune, 2012; Krych et al., 2013; Sabree & Moran, 2014; Schauer et al., 2014; Dietrich, Köhler & Brune, 2014; Perez-Cobas et al., 2015; Mikaelyan et al., 2015; Tinker & Ottesen, 2016; Zheng et al., 2017). Additionally, cultivation under oxygen-limited conditions has coaxed new species of cockroach gut bacteria into cultivation (Tegtmeier et al., 2016a, 2018).

Cockroaches are amenable to axenic rearing and such studies have helped to shed light on some possible roles of gut bacteria in cockroach development and behavior. The majority of these studies have focused on the cockroach *Blattella germanica*, reporting successful but delayed development to adulthood, with normal fertility and viability of young (House, 1949; Clayton, 1959; Benschoter & Wrenn, 1972). Host aggregation, a common cockroach behavior, was found to be stimulated by gut bacterial products in *B. germanica* that were absent when *B. germanica* was treated with antibiotics (Wada-Katsumata et al., 2015). GF *Shelfordella lateralis* cockroaches have facilitated the determination of how oxygen impacts gut tissue colonization and metabolic activity of two bacterial strains isolated from *S. lateralis* (Tegtmeier et al., 2016b). Additionally, digestive tracts in GF *S. lateralis* exposed to environmental and animal-derived inocula were capable of enriching for bacteria, primarily members of the Bacteroidetes, Firmicutes, and Proteobacteria, that were closely related to gut residents typically found in *S. lateralis* gut tissues (Mikaelyan et al., 2016). Furthermore, a clear phylogenetic signal was detected between inoculum source (i.e., cockroach > termite > mice > soil) along with recapitulation of a gut microbiota composition typically observed in *S. lateralis* (Mikaelyan et al., 2016). Although these efforts have made great strides in illustrating how cockroaches and their gut microbiota collaborate, less is known about host physiological responses to gut microbiota colonization. Recent work has shown that consumption of feces (coprophagy) from conspecifics and nestmates is common in cockroaches (Nalepa, Bignell & Bandi, 2001; Kopanic et al., 2001), and is a means for the transfer of hindgut bacteria (Rosas et al., 2018). Additionally, coprophagy provides consumers with amino acids, lipids, carbohydrates, and micronutrients that remain in the diet post-digestion and from microbes that have colonized the fecal pellet during digestion or since its deposition in the environment (Nalepa, Bignell & Bandi, 2001). Cockroach nymphs exhibit the strongest responses to aggregation pheromones present in feces (Dambach, Stadler & Heidelbach, 1995), of which at least some of these chemical signals are produced by bacteria therein (Wada-Katsumata et al., 2015), suggesting that inoculation of early-stage nymphs with gut bacteria via coprophagy is important for normal development. This study seeks to detail the impact of coprophagy on *Periplaneta americana* physiology and development, and it is expected that physiological systems within the cockroach that are most heavily influenced by exposure to microbiota-enriched frass will highlight sites of host-microbiota interactions for further study.

## Materials and Methods

### Insects

*Periplaneta americana* nymphs, adults and ootheca were obtained from a live collection maintained in the Insectary at the Ohio State University Biological Sciences Greenhouse (Columbus, Ohio).

### Ootheca treatment

Ootheca were manually detached from gravid *P. americana* females and surface sterilized by three rounds of cleaning using a detergent scrub (1% Alconox detergent), dilute bleach (0.08% sodium hypochlorite), and then an enzymatic lysis buffer (lysozyme 10 mg/ml, EDTA, Tris-HCl and Triton X-100), each step followed by three rinses with sterile MilliQ water (MQW) to remove antiseptic solution and induce intermittent hypo-osmotic shock on surface-clinging bacteria (Schwinghamer, 1980; Salema et al., 1982). An aliquot of each third MQW rinse was reserved to monitor effectiveness of previous cleanings to reduce bacterial load. Aseptic ootheca were incubated individually in sterile microcentrifuge tubes capped with sterile cotton at 31 °C for approximately 30 days until hatching, which yielded up to 16 GF nymphs per oothecum. No decreased oothecum viability due to washing treatments was observed.

### Rearing germ-free *P. americana*

Cohorts of three to four GF first instar nymphs were aseptically-transferred to sterile rearing chambers of approximately 16 cm^3^, stocked with gamma-irradiated (aseptic) rat chow and aseptic water, which was renewed weekly. Rearing chambers were passively ventilated with air filtered through a 0.22 µm membrane.

### Rearing conventionalized *P. americana*

To introduce bacteria native to *P. americana*, cohorts of three to four GF first instars were exposed to frass taken from a lab-maintained colony of non-sterile or “wild-type” (WT) *P. americana* in lieu of food for 3 days. Subsequently, conventionalized (Conv) first instar nymphs were housed under the same conditions as GF insects except for that sterility was not maintained. As each generation of *P. americana* typically acquires their gut microbial community through conspecific and nestmate coprophagy (Nalepa, Bignell & Bandi, 2001; Bell, Roth & Nalepa, 2007), the conventionalization approach used reflects the normal route of gut microbe acquisition.

### Rearing wild-type *P. americana*

One-day old first instar insects from 10 ootheca were deposited in an aquarium containing 10 adult male cockroaches from a non-sterile mixed generation colony maintained in the lab and provided with gamma-irradiated rat chow and access to MQW ad libitum; nymphs from this colony were designated “WT”. WT hatchlings were free to interact with adult cockroaches and their frass to facilitate coprophagy and subsequent acquisition of normal gut microbiota. As cannibalism of deceased nestmates is also a putative mechanism for gut microbiota acquisition, late-stage nymphs from the non-sterile colony were sacrificed and deposited in the WT colony. WT insects did not experience spatial constraints or undergo the oothecum sterilization procedure as compared to GF and Conv insects and were exposed to unfiltered air.

### Quality control

Quality control measures were employed throughout experiments to ensure maintenance of GF status and to confirm colonization of Conv individuals. Oothecum rinse water, collected between each antiseptic treatment, was plated in triplicate on Luria-Bertani agar (LBA) plates (incubated at 31 °C for up to 1 week) to detect cultivable contaminants flushed from ootheca, and to provide confidence that ootheca are aseptic as they enter incubation. Ootheca shedding no contaminants at final rinses were considered to be aseptic and used for subsequent experiments. At the time of hatching and installation into habitats, one first instar nymph from each oothecum, or the oothecum itself, was sacrificed and homogenized in sterile 1% PBS and plated on LBA in triplicate to confirm aseptic status. At the termination of insect growth, and just prior to insect dissection, frass was collected from rearing chambers, suspended in 1% PBS, and plated to confirm that habitats remained GF throughout the duration of the experiment. Bi-monthly sampling of GF-reared individuals for contamination was performed by homogenizing individuals at various instars and plating homogenate on LBA. During sterile treatment method development, diagnostic PCR with universal 16S primers 27F (5′-AGAGTTTGATCMTGGCTCAG-3′) and 1391R (5′-GACGGGCGGTGTGTRCA-3′) was performed on DNA extracted from homogenates prepared from GF insects and their frass to detect non-cultivable contaminants of the gut or habitat. Additionally, microscopic examination of 4′,6-diamidino-2-phenylindole (DAPI)-stained frass, gut contents, and gut thin-sections were performed to further evaluate the effectiveness of the GF, and conventionalization protocols.

### Instar duration measurement

Germ-free and Conv nymphs raised in cohorts of three to four insects were monitored individually for molting activity, and dates of instar transitions of individuals within each cohort were recorded. Aquarium rearing of WT insects in cohorts of dozens of individuals prevented tracking of individual insects and corresponding molt date and developmental times within the WT treatment. Insects dissected at subsequent instars for gut morphological measurements resulted in decreasing sample sizes at later instars, and non-parametric statistical methods were used when making comparisons across instars. Targeting early instar insects constrains the experiment to a relatively short timeframe, as rearing to adulthood is prohibitively long (6–12 months for WT insects), with GF *P. americana* progression to adulthood uncertain.

### Morphological measurements

Nymphs were collected as they molted to third, fourth, and fifth instars, and duration (days) of these instars were recorded. Previous work has shown that the cumulative effects of bacterial colonization, or lack thereof, in the host could be observed in fourth and fifth instars and thus all experimental measures in this study were taken from fifth instar individuals (Bracke, Cruden & Markovetz, 1978; Gijzen & Barugahare, 1992). Nymphs at designated life stages were dissected approximately 5 days after molting, at peak of feeding within instar (Valles, Strong & Koehler, 1996), to minimize variability in gut morphology associated with instar transitions. Eight morphological metrics were collected in millimeters (mm), unless otherwise noted: body length, body width, body mass (grams, g), whole gut length, foregut length, midgut length, hindgut length, and gut mass (g). FIJI image analysis package (ImageJ) was used to perform measurements of the full length of dissected guts (esophagus to anus) and their corresponding carcasses from digital images of these tissues taken immediately following dissection. Results of statistical tests performed on comparisons of morphological measurements are reported in Table S1. Additionally, gut compartments and bodies were traced and lengths measured, with measurements calibrated to a scale in each photo. Mass measurements were also collected for whole GF, Conv, and WT individuals prior to dissection and of their dissected digestive tracts using a microbalance. Additionally, qualitative observations of gut texture, color, opacity, and segmentation were collected from dissected fifth instar GF, Conv, and WT individuals.

### Phenotype analysis

All statistics were performed in R using vegan and FSA packages (Ogle, Wheeler & Dinno, 2018; R Development Core Team, 2013; Oksanen et al., 2015). Morphological measurements at instar rather than calendar age yielded differences in sample size, as insects aged out of instar classes at different rates; non-parametric statistics were utilized to accommodate sample size variation. Instar duration, and morphological measurements were analyzed for statistical differences using a Mann–Whitney Test for pairwise-comparisons. Kruskal–Wallis and Dunn Tests were performed for multiple comparisons, with Bonferroni correction-adjusted *p*-values. Principal component analysis (PCA) was performed to simultaneously examine multiple morphological variables across treatments. The function “Varpart” within the vegan R package was used to partition variance among morphological variables and the PCA was constrained to the three variables representing 93% of the variation. Subsequently, multi-response permutation procedure (MRPP) was used to assess the significance of the observed differences between treatment centroids.

## Results

### Germ-free insects exhibited reduced width and prolonged instar duration

**Figure 1:**
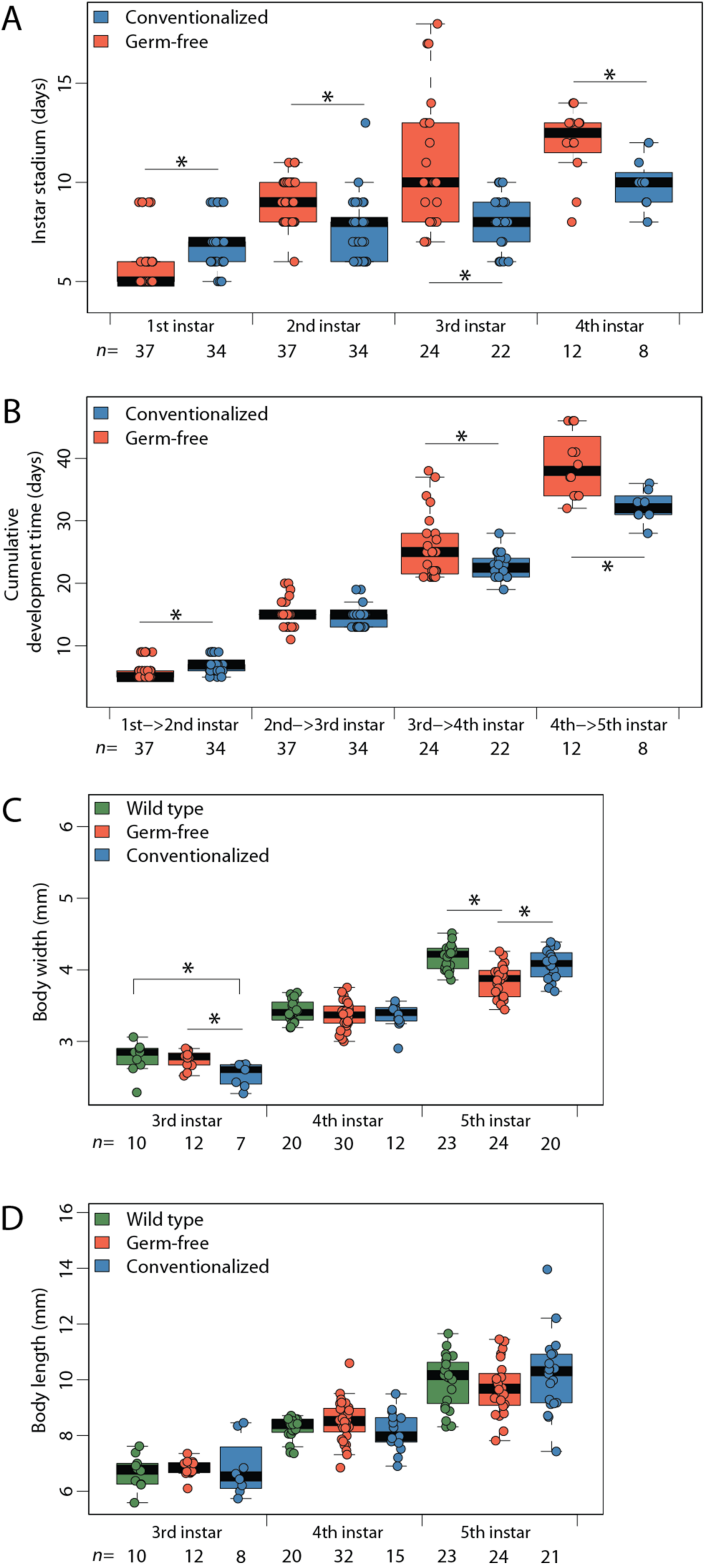
Maturation rate and body morphology. Limiting access to gut bacteria via coprophagy prolongs development in *Periplanata americana*. (A) Duration (stadium) of individuals within each life stage; (B) Cumulative development time; (C) Body width measurements; (D) Body length measurements. Asterisks indicate significant (*p* < 0.05) differences given a Dunn test. n, number of individuals measured per sample.

Germ-free (*n* = 37) *P. americana* individuals remained in each instar an average of two days longer than Conv (*n* = 34) individuals (Fig. 1A), which resulted in longer times between molts in GF insects and prolonged developmental periods (Fig. 1B) being observed. While Conv insects molted to fifth instar after an average of 32.3 days, GF insects required an average of 38.9 days to reach the same life stage. While stadium duration of WT insects was not obtained for every instar because of the complexity of tracking individual insects in a large cohort, average age at fifth instar was 30.2 days (Fig. S1). GF insects also exhibited the lowest average body width (3.84 mm) when compared to WT (4.18 mm) and Conv (4.08 mm) insects (Fig. 1C). Body lengths of GF and Conv individuals were not significantly different at third, fourth, or fifth instars (Fig. 1D), and body mass did not differ between treatments (Fig. S2).

### Conventionalization of germ-free *P. americana* recovers normal hindgut tissue morphology and development

**Figure 2:**
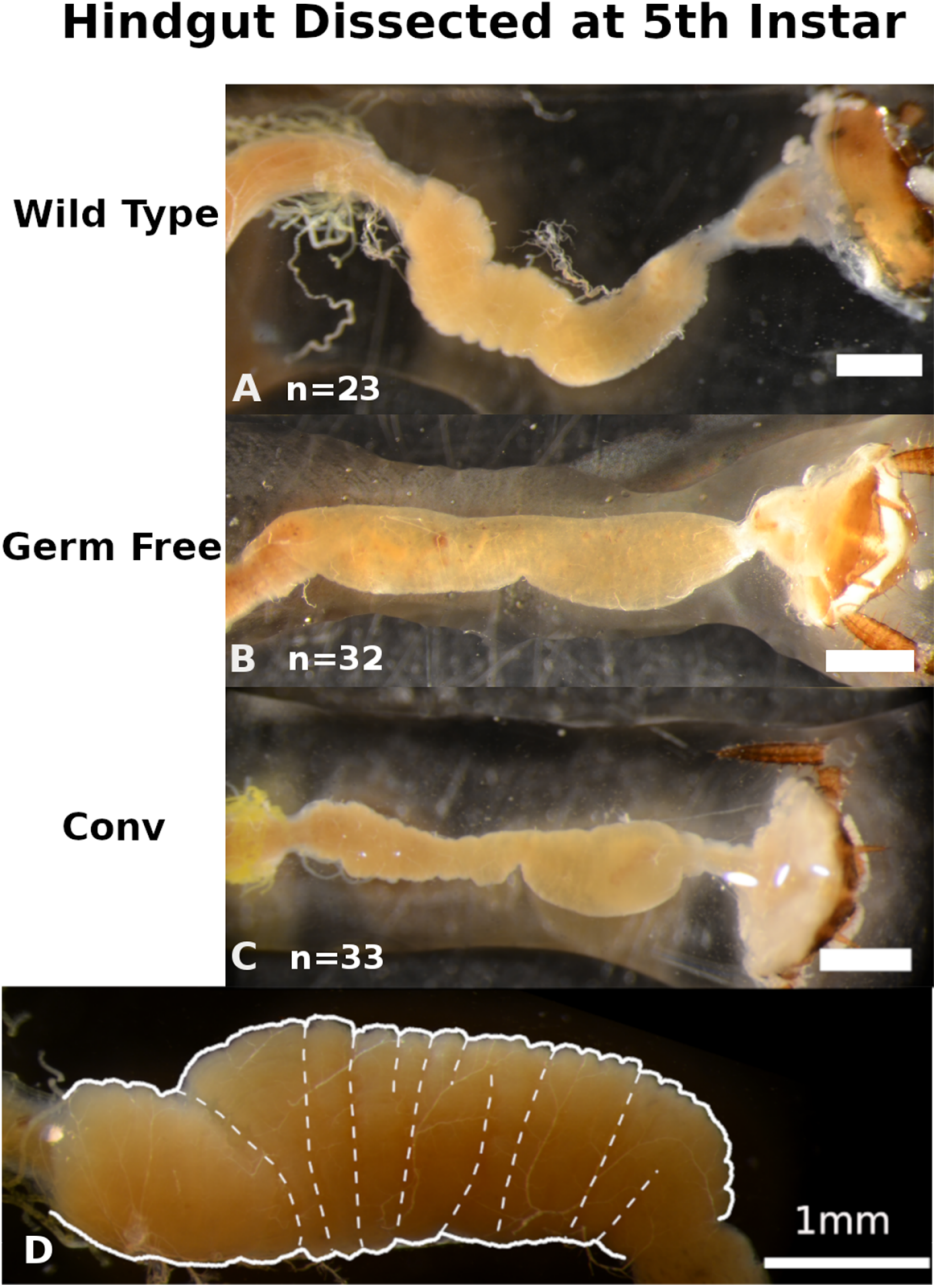
Qualitative comparisons of hindgut morphology in fifth instar *Periplaneta americana*. Exemplars from three wild-type (A), germ-free (B), and conventionalized (Conv.; C) *P. americana* individuals are presented. A magnified hindgut from a wild-type *P. americana* (D) is provided to highlight normal morphological features, including undulating gut margin highlighted by a thick white line and gut segmentation highlighted with thin dashed lines. Scale represents one mm.

**Figure 3:**
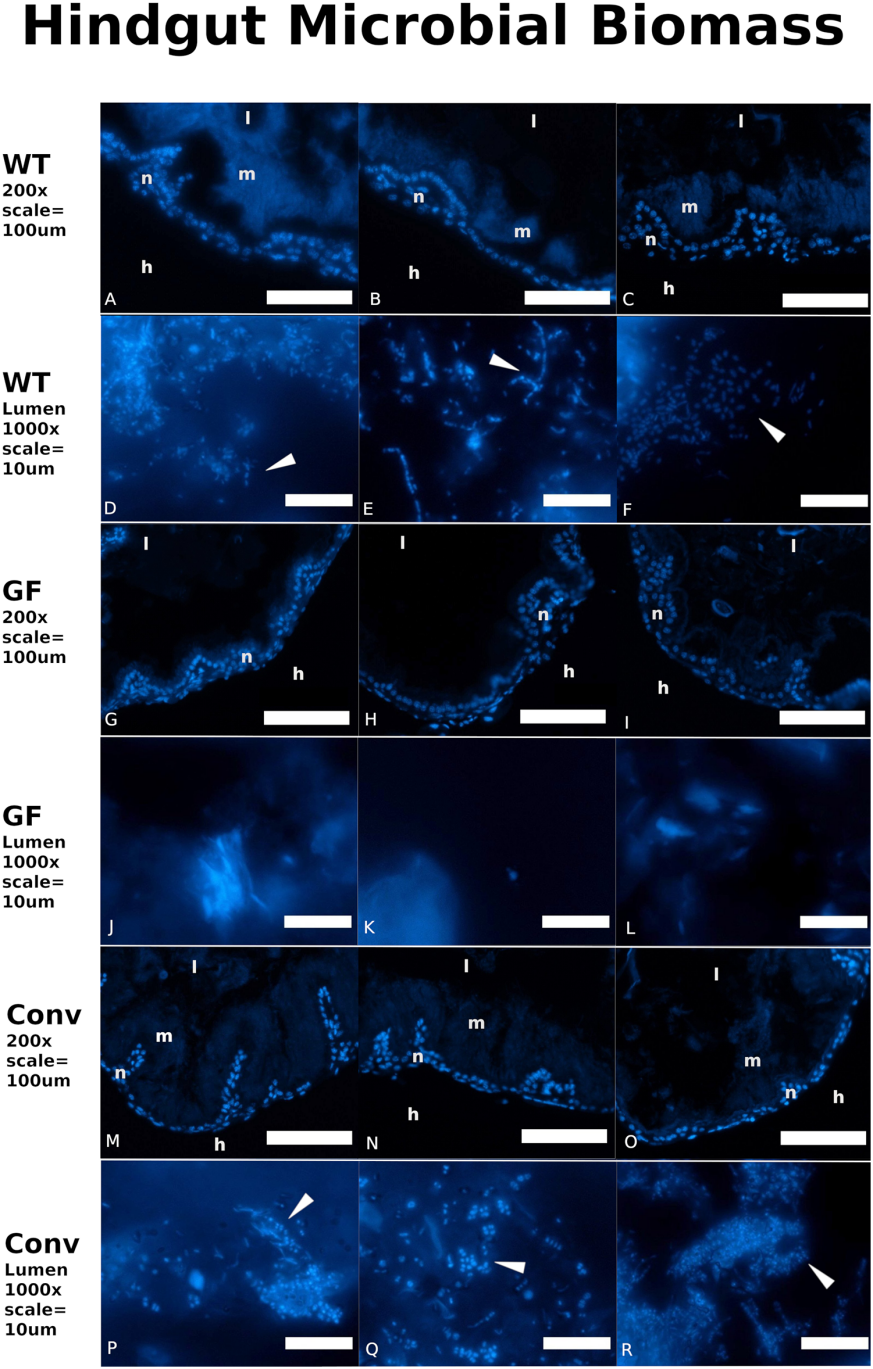
Conventionalized *Periplanata americana* hindguts are enriched with microbial biomass. Wild-type and conventionalized *Periplanata americana* hindguts are enriched with microbial biomass that aggregates adjacent to the epithelium, while germ-free hindguts lack microbial biomass. 4′,6-diamidino-2-phenylindole (DAPI) stained thin-sections of hindguts from wild-type (WT) (A–F) (*n* = 3), germ-free (GF) (G–L) (*n* = 3) and conventionalized (Conv) (M–R) (*n* = 3) *P. americana* were viewed under epifluorescence to visualize DNA associated with epithelium and luminal contents. h, Hemocoel; l, lumen; n, gut epithelial cell nuclei; m, microbial biomass; arrows—fluorescence consistent with bacterial morphology.

Distinct visual contrasts between fifth instar WT (*n* = 23) (Fig. 2A; Figs. S3A–S3C) and GF (*n* = 32) (Fig. 2B; Figs. S3D–S3F) hindgut tissues were consistently evident. WT *P. americana* hindguts are characterized by numerous lateral folds along the length of the hindgut that are visible as an undulating gut margin with lateral creases (Fig. 2D). All inspected WT hindguts were opaque, turgid in texture, and generally filled with mustard-yellow hindgut contents (Fig. 2A; Figs. S3A–S3C). Conv hindguts (Fig. 2C; Figs. S3G–S3I) were similar to those from WT insects in that they were turgid in texture and more opaque than GF hindguts (Fig. 2B; Figs. S3D–S3F), and Conv hindguts exhibited frequent lateral folds and coloration characteristic of WT insects. Further, dissection of Conv revealed mustard-yellow contents, similar to those observed in WT digestive tracts, and this appears to be a mix of digestate and bacterial biomass (note the fluorescence of material in gut lumen in Fig. 3) lodged in hindgut folds (Fig. 3). DAPI stained hindgut tissue thin sections reveal a dense mat of bacterial biomass adjacent to the host gut epithelial lining and planktonic bacterial growth deep within the lumen of WT and Conv insects (Fig. 3). In contrast, GF hindguts were uniformly flaccid, translucent, and displayed less frequent and less pronounced lateral folding (Fig. 2B; Figs. S2D–S2F). Additionally, gut contents were comprised of partially digested diet and no evidence of bacteria were observed (Fig. 3). No amplicon was observed after diagnostic PCR conducted on GF frass DNA extract (Fig. S4) using bacteria-specific primers. Despite thorough cleaning of fat bodies from guts at the time of dissection, the fat body specific endosymbiont *Blattabacterium* was detected in diagnostic PCR reactions of gut tissue DNA extracts from GF insects, negating the utility of bacteria-specific primers for contamination detection in gut homogenates by diagnostic PCR.

**Figure 4:**
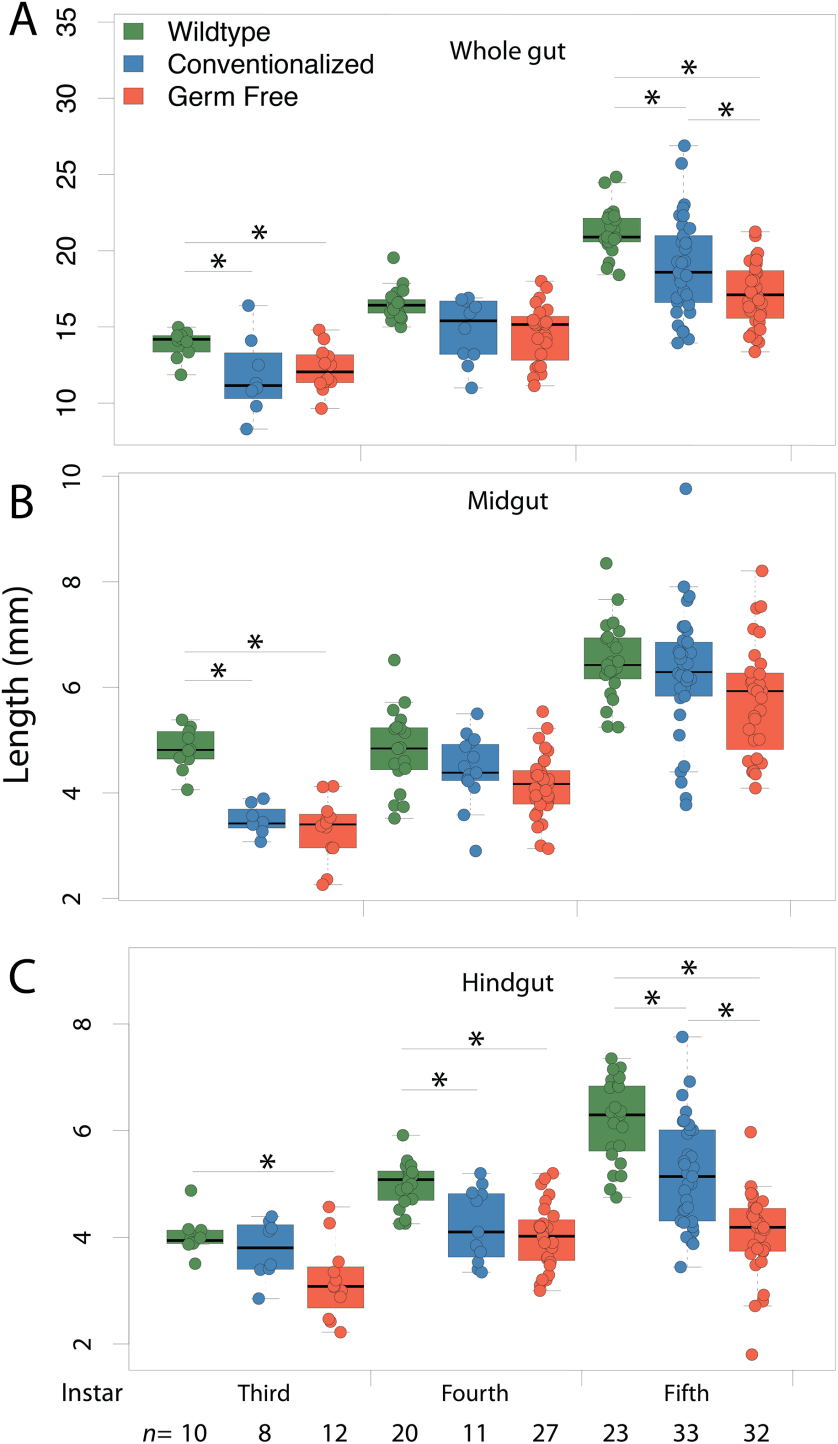
Gut morphology. Conventionalization of germ-free *Periplaneta americana* partially recovers wild-type gut length morphology as individuals age. Whole gut (A) midgut (B) and hindgut (C) lengths were measured in germ-free (GF), conventionalized (Conv) and Wild-Type (WT) individuals at third, fourth and fifth instars, and, at the sixth instar for WT and Conv individuals. Asterisks indicate significant (*p* < 0.05) differences given a Dunn test. n, number of individuals measured per sample.

Average length of the complete fore-to-hindgut tissues was shorter in GF insects (17.13 mm) than in both Conv (18.96 mm) and WT (21.24 mm) insects (Fig. 4A; Dunn Test: GF-Conv *p* = 0.0244, Conv-WT *p* = 0.0028, GF-WT *p* = 0.0054). When fore- (Fig. S5) and mid-gut (Fig. 4B) sections were examined separately, average lengths did not differ significantly between any treatments at any instar, except for between GF (3.30 mm) and either WT (4.83 mm) or Conv (3.48 mm) third instar midguts (Dunn Test: WT-GF *p* = 0.0058, Conv-GF *p* = 0.0455). Conversely, hindgut length was significantly reduced in GF insects at third, fourth, and fifth instars relative to either Conv or WT insects (Fig. 4C). Finally, significant differences in gut mass were only detected at fourth instar between GF and WT insects (Fig. S6), with respective masses of 5.2 and 6.7 mg (Dunn Test: WT-GF *p* < 0.0070), but variability was high within treatments.

### Wild-type, conventionalized, and germ-free insects bear morphological signatures that define a gradient across treatments

**Figure 5:**
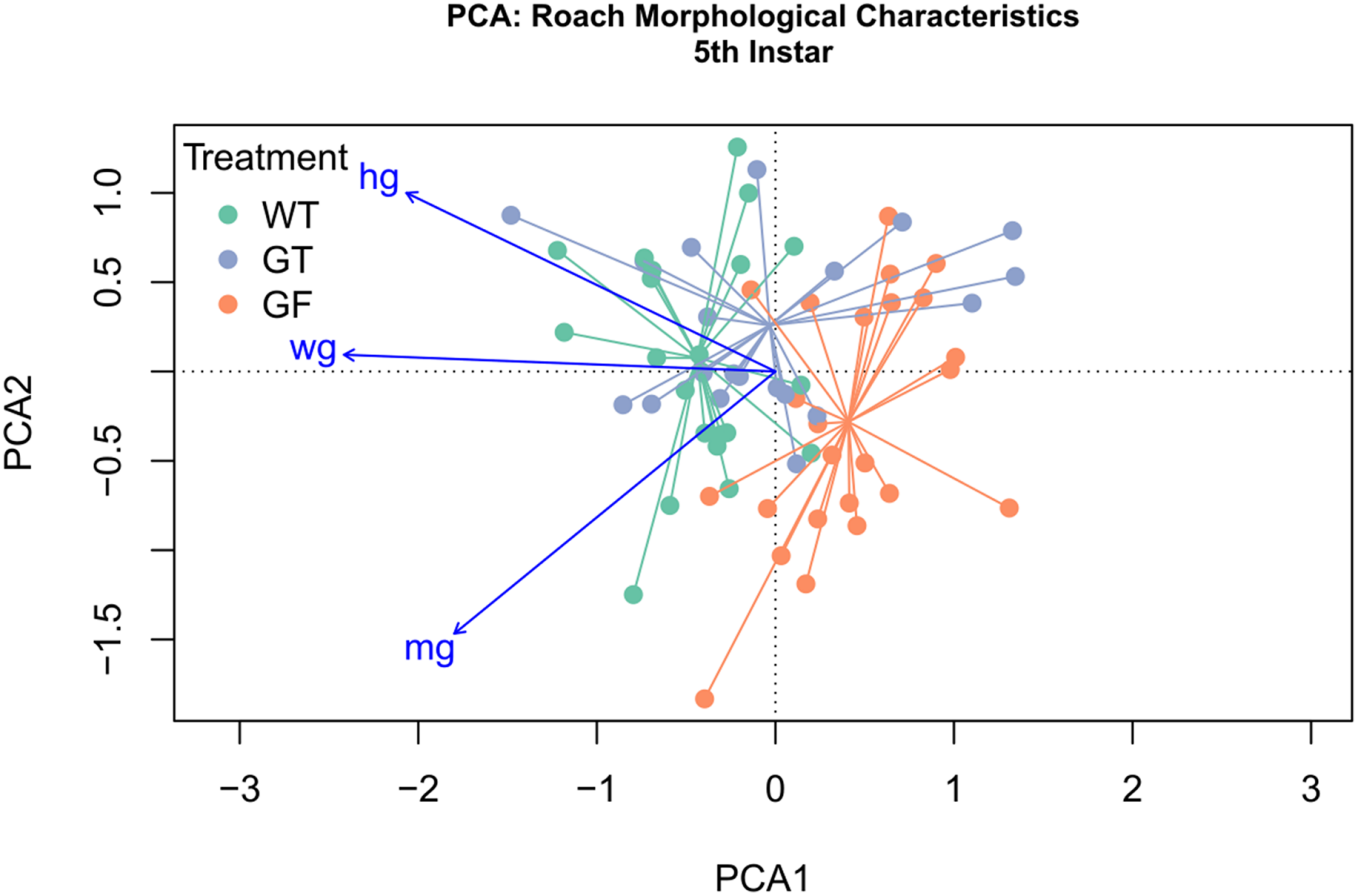
Principal Component Analysis (PCA) of morphological characteristics at fifth instar. PCA and ordination was performed, constrained by the three variables explaining the greatest variation, specifically, whole gut length (wg), hindgut length (hg), and midgut length (mg). The first component explained 84.5% of variation, with 6.9% and 2.2% explained by the second and third components. Treatment clusters displayed the least overlap at instar 5 and are situated with Conventionalized (Conv) treatment sandwiched between germ-free (GF) treatments and Wild-Type (WT). Distance between treatment centroids was 0.22 between the WT and GF centroids, 0.15 between the Conv and GF centroids, and 0.12 between the Conv and WT centroids. All vector loadings are significant (*p* < 0.000999) with *r*^2^ values of 0.99 (wg), 0.91 (hg), and 0.89 (mg). Vector length is proportional to fit.

Of eight morphological variables examined in this study whole gut length, midgut length, and hindgut length explained 91.4% of variance across treatments (Fig. S7). PCA was performed to examine the relationship among treatments at each instar in reference to these three response variables. Ordination was conducted on the first two principal components, representing 84.5% and 6.9% of variance explained, respectively (Fig. 5). Treatment centroids were significantly different (MRPP: *p* < 0.001), with the Conv centroid being intermediate to that of GF and WT centroid. All vector loadings associated with morphological variables were significant, with strong linearity (*p* < 0.001, whole gut length *r*^2^ = 0.99, midgut length *r*^2^ = 0.91, hindgut length *r*^2^ = 0.89). Loading vectors for hindgut length and whole gut length demonstrate a gradient of low values in proximity to the GF centroid to high values toward the WT centroid. The vector for midgut length appears to follow a gradient associated with within-instar variation, as midgut length varies more within instar than across treatments. A second PCA was performed on data from all instars and treatments, simultaneously, with 93.1% and 1.9% of variance explained by the first two axes, yielding greater linearity of loading vectors but reduced separation of centroids (Fig. S8). With multiple instars represented within each treatment, vector loadings previously associated with within-instar variation (i.e., midgut length) realign to describe an across-treatment gradient, implying that variation in midgut length is better explained by treatment than instar or within-instar effects.

## Discussion

We demonstrated that GF *P. americana* exposed to frass from lab-reared, WT conspecifics partially restored instar duration periods and digestive tissue development, particularly in hindgut tissues, to levels observed in WT insects, and these findings suggest a relationship between exposure to gut microbiota in cockroach frass and host digestive tissue and developmental outcomes. *P. americana* and other cockroaches typically live in dense multigenerational aggregates that afford them easy access to waste, discarded exoskeletons, and dead bodies, and the microbes therein, of conspecifics (Mira, 2000; Nalepa, Bignell & Bandi, 2001; Kopanic et al., 2001; Rychtář et al., 2014). These social and dietary behaviors are common in cockroaches and reflect a reliable means for the transmission of gut microbes that contribute to host fitness (Nalepa, Bignell & Bandi, 2001; Sabree & Moran, 2014). We hypothesized that *P. americana* relies upon community-acquired gut microbes for normal development, and preventing their exposure to these microbes (as in GF insects) would result in growth and developmental defects. GF insect growth stalled in fourth and fifth instars, as no insects progressed to sixth instar within the timeframe of the experiment and some insects remained in fifth instar indefinitely. Future work may determine whether germ free *P. americana* progress to adulthood. GF insects conventionalized by a single exposure to conspecific frass early in life (i.e., Conv insects) exhibited a partial, but significant, remediation of instar duration (Figs. 1A–1C) and gut tissue development (Figs. 2A–2D, 3 and 4A–4C). Interestingly, Conv insects exhibited an initial delay in growth and maturation at first instar, compared to both WT and GF insects (Figs. 1A–1D). This was likely due to the 3 day exposure to frass, without food, to enforce frass consumption. Yet, the cumulative benefit of microbial colonization allowed the Conv insects to surpass the GF and approach growth and maturation levels observed in WT insects by fifth instar. Given these data, it was surmised that the observed positive growth and developmental responses to frass exposure and subsequent gut colonization were cumulative due to persistent colonization of the cockroach gut rather than a burst of growth at time of exposure. It is notable that *Blattabacterium* was not removed as part of making GF insects and was present in GF, Conv, and WT insects, yet its contributions alone did not support normal host development in the GF insects.

The partial recovery of WT growth and development observed may be due to Conv insects having received only a single exposure to conspecific frass, instead of multiple exposures, during development. Specifically, Conv insects were restricted to a single exposure to WT frass during their first instar and were housed in small cohorts (*n* < 4) for the duration of the experiment (i.e., through fifth instar) and each experienced at least four moltings. Molting represents a potential bottleneck event during which portions of the gut lining, along with substantial gut contents and associated bacteria, are shed (Engel & Moran, 2013). If the exuvium and gut lining are not rapidly consumed by insects there is potential for loss of gut microbes, especially oxygen-sensitive taxa (e.g., some members of the *Bacteroidetes* and *Firmicutes*), that may not easily be horizontally reacquired through coprophagy within a relatively tiny cohort of insects of the same life-stage experiencing the same experimental conditions. Under normal conditions, developing insects living in multigenerational, population dense communities would have several opportunities to reacquire gut bacteria lost as a result of molting by coprophagy. As these experimental conditions were meant to severely limit exposure to microbes present in frass, further work is planned to determine minimum frass exposure frequency for nymphs to achieve WT growth and development.

An additional explanation for the observed results is that the absence of gut microbes in GF insects may have limited the host’s access to dietary nutrients that are typically liberated by members of the gut microbiota, and thus prolonged their development due to inadequate access to nutrients. Cockroaches and other insects that consume a diet comprised primarily of plant biomass that is rich in recalcitrant polysaccharides like pectin, cellulose, and hemicellulose have a characteristically enlarged hindgut colonized with diverse microbes that serves as an anaerobic digester for these biopolymers (Bignell, 1977, 2016; Breznak, 1982; Kane & Breznak, 1991; Kane, 1997; Bell, Roth & Nalepa, 2007; Watanabe & Tokuda, 2010). Exposure to conspecific frass partially recovered WT levels of gut tissue development and instar duration, which may indicate the colonization of some endemic taxa that facilitate dietary nutrient accessibility. It was notable that despite little size and weight difference at equivalent instar, the gastrointestinal tract developed differentially in GF and Conv insects, especially in the hindgut where the highest numbers of bacteria have been found. The hindgut is a major site of microbial activity along the cockroach alimentary tract, with the greatest numbers of bacteria consistently identified in this compartment through methods as diverse as microscopy, cultivation, and 16S sequencing (Bignell, 1977; Bracke, Cruden & Markovetz, 1979; Schauer, Thompson & Brune, 2012; Schauer et al., 2014). As such, it is unsurprising to find significant divergence in growth phenotype between GF and either WT or Conv insects in this compartment in the absence of microbial influence. Additionally, the microbial and biochemical composition of the frass is likely more similar to that of the hindgut than the midgut, which may explain why the remediative value of the frass was more pronounced in these tissues.

Gut microbes have been shown to mediate nutritional and immune factors and influence physiological systems (Engel & Moran, 2013), and numerous avenues to microbial promotion of host growth have been identified in other model organisms, and are likely functioning in *P. americana*. Efforts to link gut microbiota to host health and development have been spurred by the tremendous progress being made to detail the membership and composition of these communities. Model systems, especially GF animals, provide invaluable platforms for linking microbes to specific host outcomes. *P. americana* is a valuable addition to available invertebrate model systems because it harbors species of many bacterial taxonomic groups typically found in some vertebrates, including humans, is trophically omnivorous, and can be reared GF without antibiotics and, given this study, responds positively when exposed to conspecific frass. The relatively low rearing costs and high fecundity add to the amenability of the GF *P. americana* model system, which further ensures that many of the questions raised by results obtained in this study can easily be experimentally pursued. Further efforts to scrutinize how conspecific frass and its biochemical and microbial components are linked to host growth and development are underway and may reveal details of host-microbe interactions that may be generalizable to other animals.

## Conclusion

A single inoculation of germ free *P. americana* with conspecific frass was sufficient to yield persistent gut colonization by microbiota, significantly decrease instar duration and speed maturation to fifth instar, while significantly increasing hindgut length. Future work should examine whether inoculation frequency further impacts development.

## Supplemental Information

10.7717/peerj.6914/supp-1 Supplemental Information 1 File S1. This file includes three bar charts summarizing various body mass, foregut length, and gut mass comparisons between germ-free, wild-type, and conventionalized *P. americana*. Additionally, a Venn diagram describing variation attributed to each morphological parameter and a Principal Components Analysis of treatment groups. Finally, a table summarizing the results of statistical comparisons of the treatment groups.

10.7717/peerj.6914/supp-2 Supplemental Information 2 Raw data for Morphology Boxplot and PCA. Raw data includes morphological measurements such as body mass (bm), body width (bw), body length (bl), gut mass (gm), whole gut length (wg), foregut length (fg), midgut length (mg), and hindgut length (hg) along with categorical variables associated with treatment, oothecum cohort, and instar.

10.7717/peerj.6914/supp-3 Supplemental Information 3 Raw data for Instar Duration and Molt Date Boxplot. Raw data includes duration of days spent in each instar in the column durval and cumulative days to each molt in the column moltval along with categorical variables associated with instar and treatment.

10.7717/peerj.6914/supp-4 Supplemental Information 4 Raw data R scripts for PCA. Contains R scripts for PCA at each instar and a PCA at all instars along with code for ordinations.

10.7717/peerj.6914/supp-5 Supplemental Information 5 Raw data R code for Instar Duration and Time to Molt boxplots. File contains R code for generating instar duration boxplots and time to 4th molt plots.

10.7717/peerj.6914/supp-6 Supplemental Information 6 Raw data R code for Morphology boxplots. File includes R code for generating boxplots to summarize morphological data.

## Additional Information and Declarations

### Data Availability

The following information was supplied regarding data availability: Raw measurements are available in Data S1 and Data S2. Supplementary Files Code S1, Code S2, and Code S3 include the R code used to generate figures and statistics.
