# Supplementary material for: Conspecific coprophagy stimulates normal development in a germ-free model invertebrate
Source: PeerJ. 2019 May 13;7:e6914. doi: 10.7717/peerj.6914 (PMC6521811; doi:10.7717/peerj.6914)
Supplement: Supplemental Information 1 — This file includes three bar charts summarizing various body mass, foregut length, and gut mass comparisons between germ-free, wild-type, and conventionalized P. americana. Additionally, a Venn diagram describing variation attributed to each morphological parameter and a Principal Components Analysis of treatment groups. Finally, a table summarizing the results of statistical comparisons of the treatment groups. [file peerj-07-6914-s001.docx]

**Supplemental Materials (S1)**

**SUMMARY**

This file includes three bar charts summarizing various body mass, foregut length and gut mass comparisons between germ-free, wild-type and conventionalized *P. americana.* Additionally, a Venn diagram describing variation attributed to each morphological parameter and a Principal Components Analysis of treatment groups. Finally, a table summarizing the results of statistical comparisons of the treatment groups.

**Supplemental Figure and Table Legends**

Fig. S1 Age at 5^th^ instar of wild-type insects for reference to germ-free and conventionalized insects.

Fig. S2 Body mass comparisons between wild-type, germ-free, and conventionalized *P. americana* at four life stages. Asterisks indicate significant (p<0.05) differences given a Mann-Whitney test. n= number of individuals measured per sample.

Fig. S3 Qualitative comparisons of hindgut morphology in 5th instar Periplaneta americana. Exemplars from three wild-type (A-C), germ-free (D-F), and conventionalized (Conv.; G-I) P. americana individuals are presented. A magnified hindgut from a wild-type P. americana (J) is provided to highlight normal morphological features, including undulating gut margin highlighted by a thick white line and gut segmentation highlighted with thin dashed lines. Scale represents 1mm.

Fig. S4 dPCR of DNA extracts from GF frass showing no amplification using universal primers among GF samples and amplification in one contaminated sample, in addition to the positive control.

Fig. S5 Foregut length comparisons between wild-type, germ-free, and conventionalized *P. americana*. Asterisks indicate significant (p<0.05) differences given a Dunn test. n= number of individuals measured per sample.

Fig. S6 Full-length gut mass comparisons between wild-type, germ-free, and conventionalized *P. americana* at fourth instar. Asterisks indicate significant (p<0.05) differences given a Dunn test. n= number of individuals measured per sample.

Fig. S7 Partition of variation among morphological parameters. While eight morphological parameters were evaluated, three variables explained 93% of variation within the data; whole gut length, hindgut length, and midgut length.

Fig. S8 Principal component analysis (PCA) of morphological characteristics at all instars constrained by the three variables explaining the greatest variation, specifically, whole gut length (wg), hindgut length (hg), and midgut length (mg). The first component explained 93.1% of variation, with 1.9% and 0.9% explained by the second and third components. Distance between treatment centroids was 0.12 between the WT and GF centroids, 0.078 between the Conv and GF centroids, and 0.053 between the Conv and WT centroids. All vector loadings were significant (p<.000999) with r2 values of 0.99 (wg), 0.93 (hg), and 0.94 (mg). Vector length is proportional to fit.

Table S1 Summary Stats. The Kruskal-Wallis Test was used to determine significant differences among multiple treatment comparisons. A Dunn-Test followed significant results of multiple comparisons yielding significant differences among pairwise comparisons. In the few cases where samples for only two treatments were available, results for a Mann-Whitney Test are reported^1^.


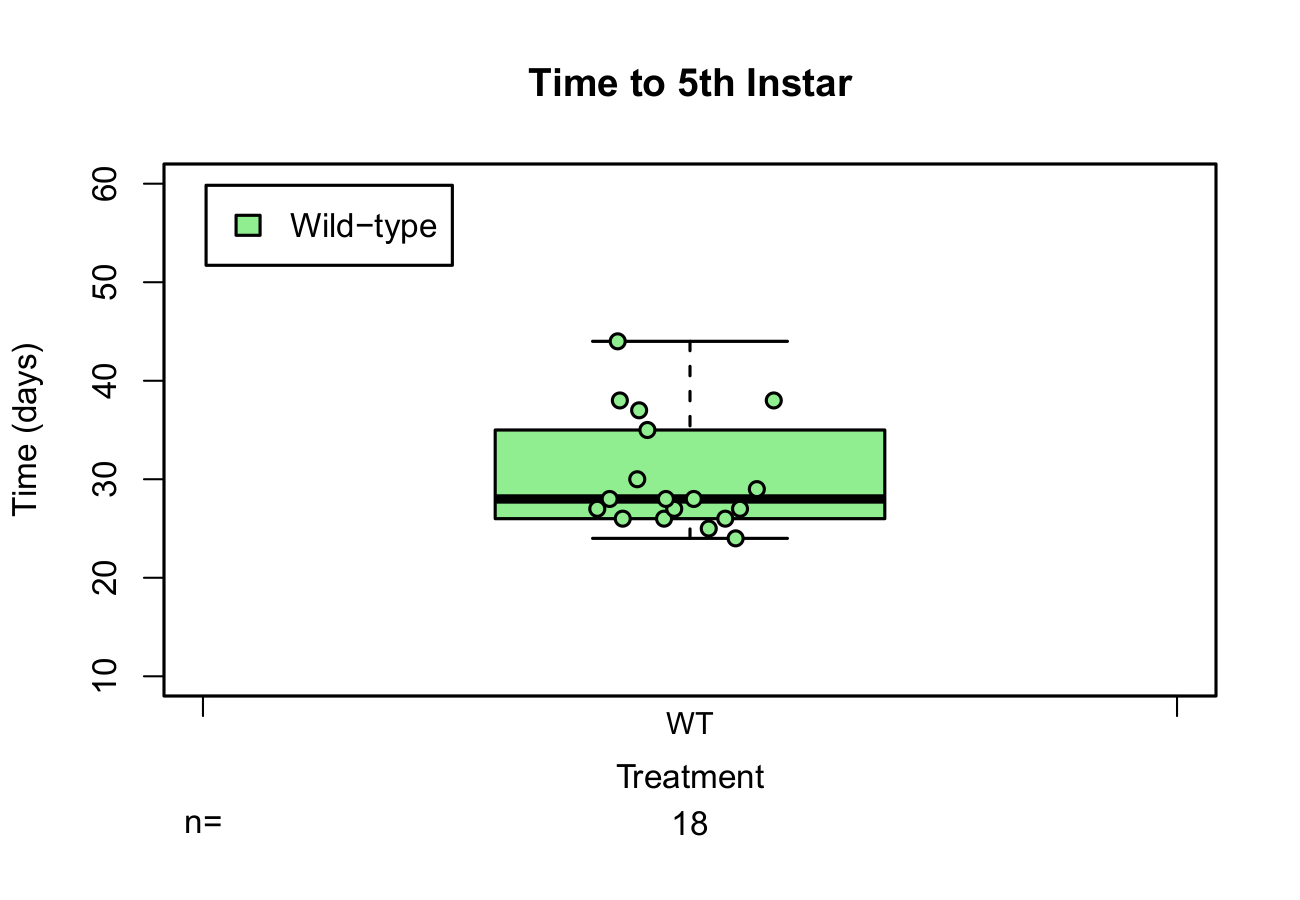


Figure S1


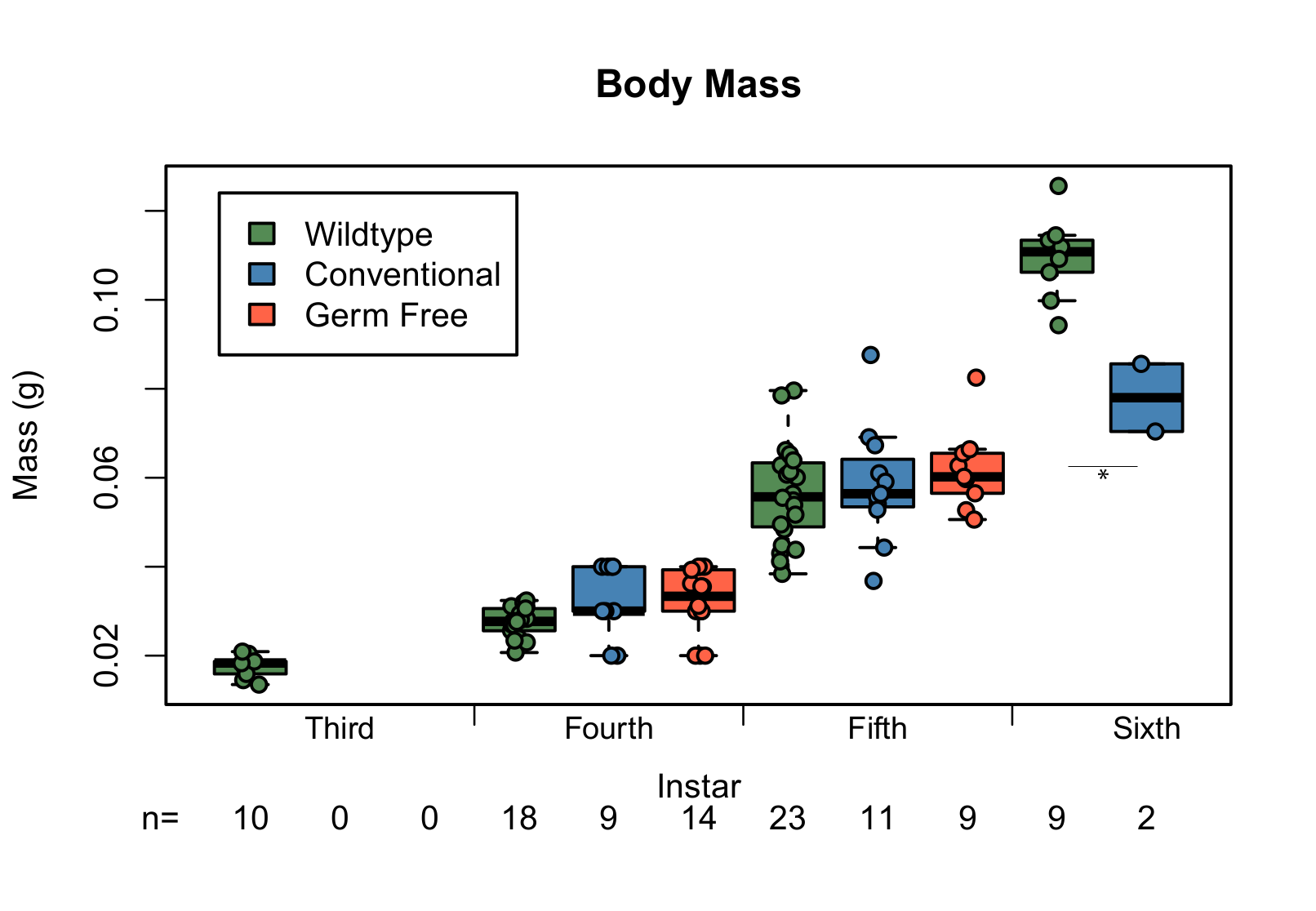


Figure S2


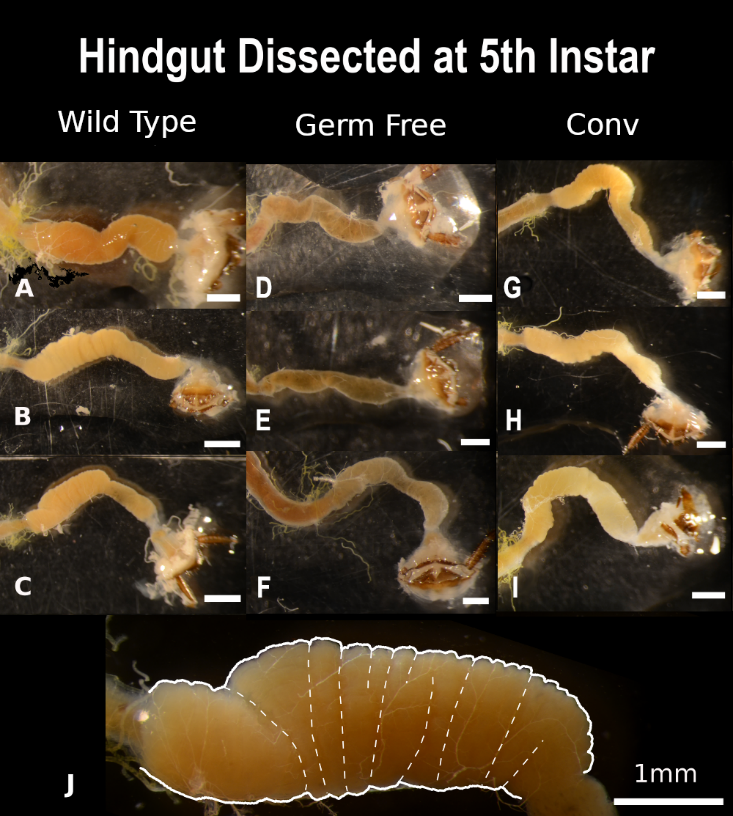


Figure S3


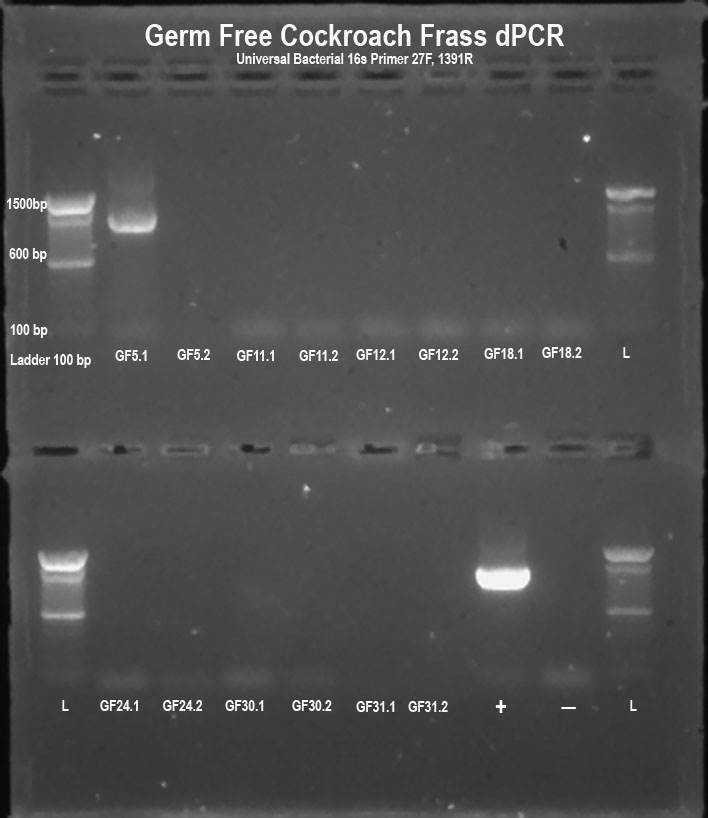


Figure S4


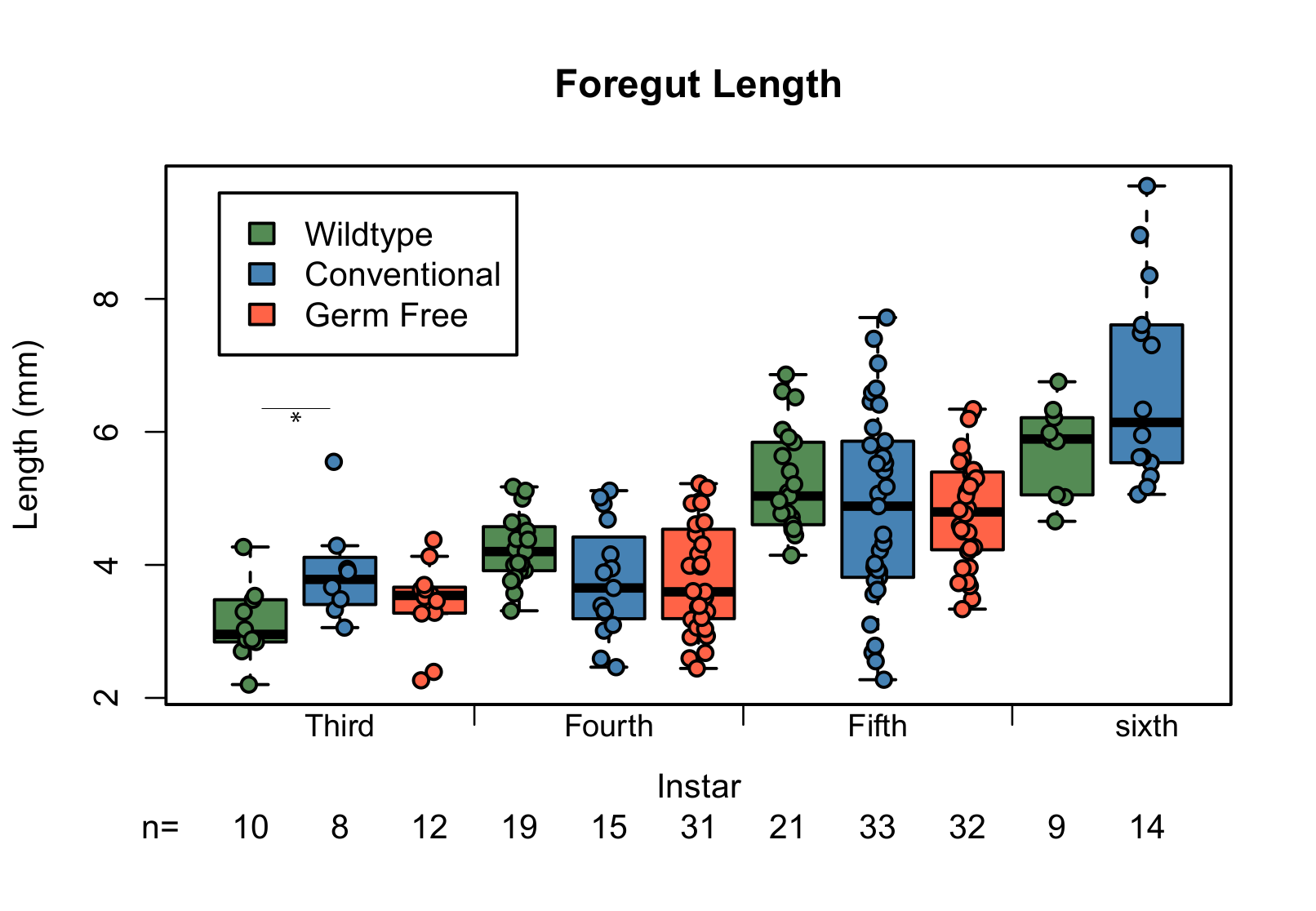


Figure S5


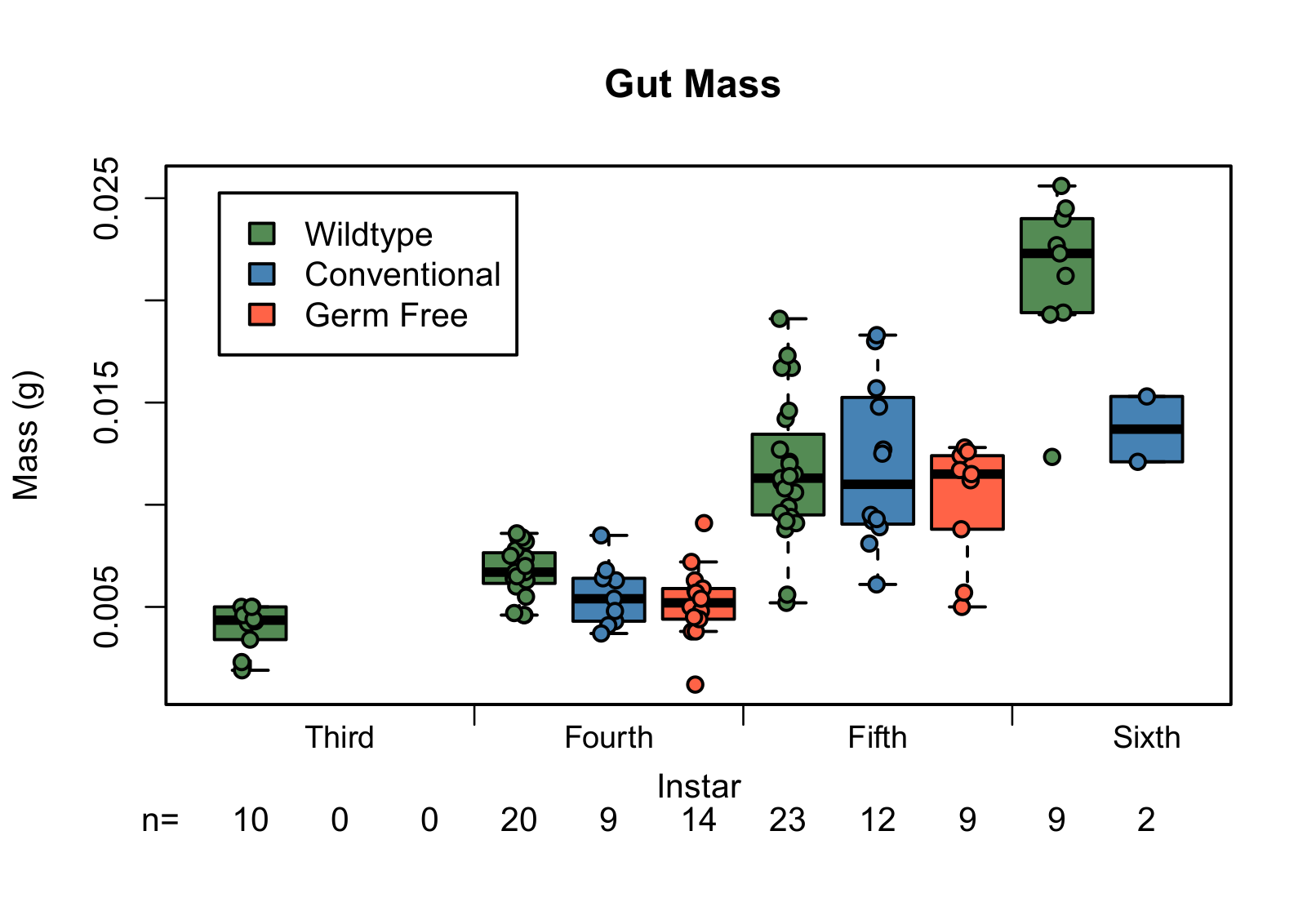


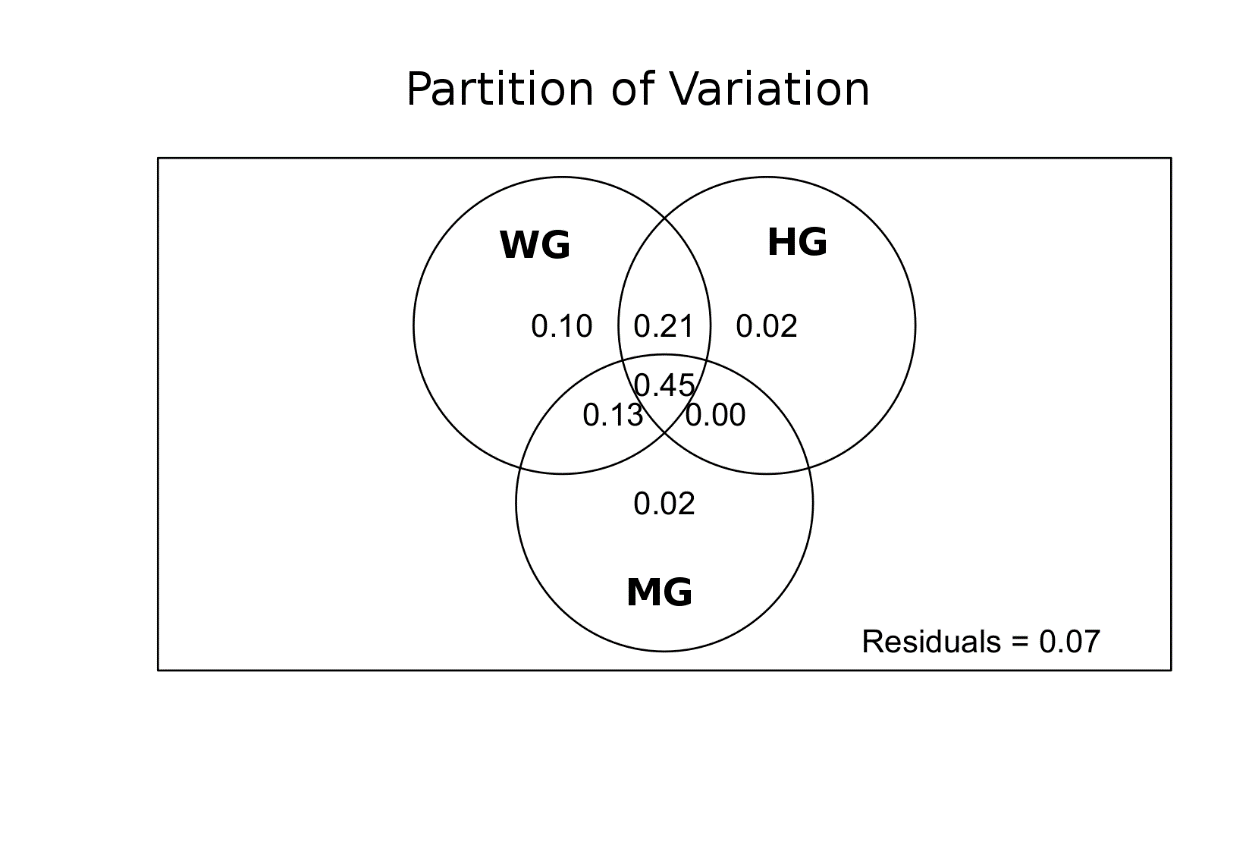
Figure S6

Figure S7

Figure S8


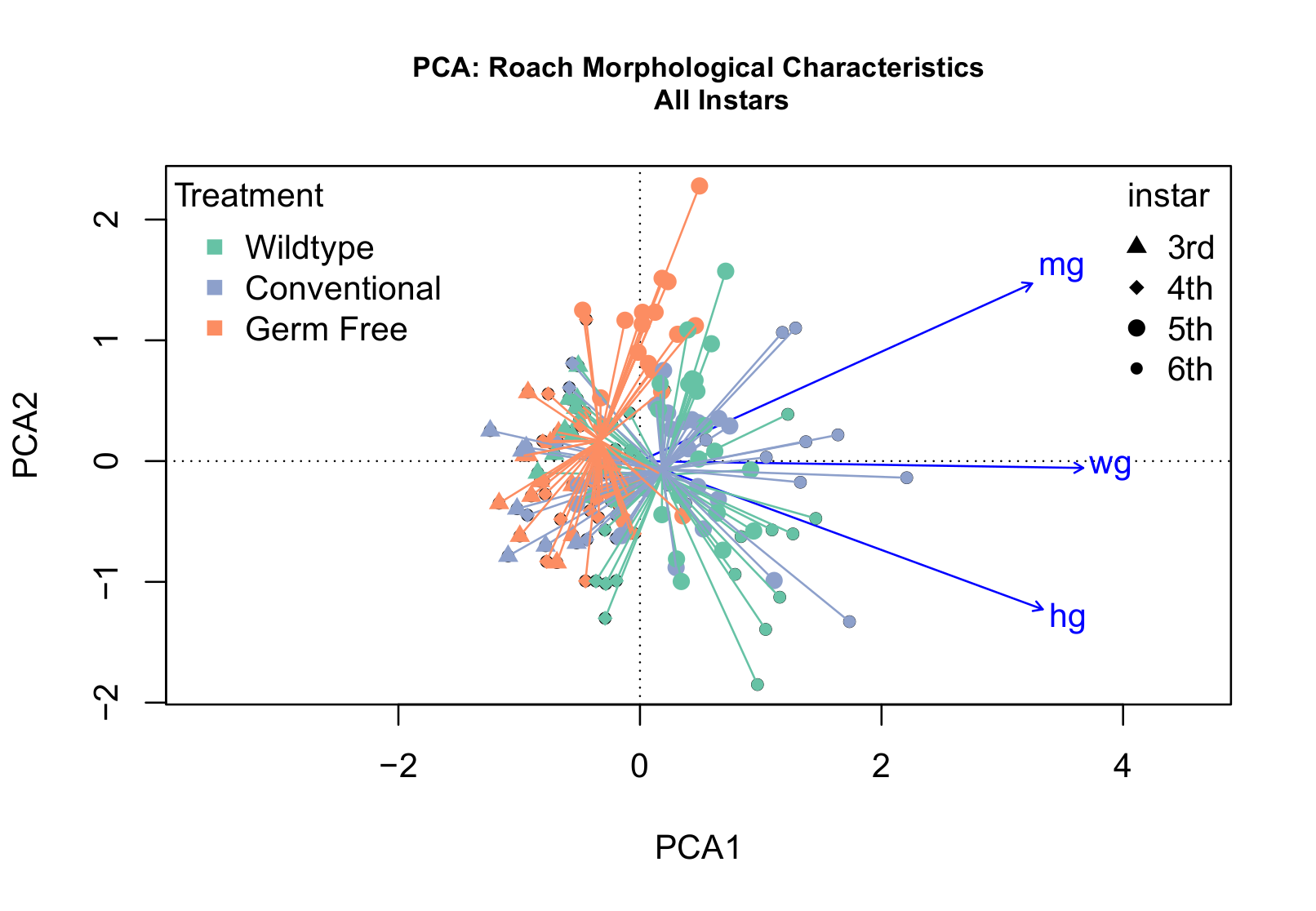


| Instar | comparison | Body Length | Body Width | Whole Gut | Foregut | Midgut | Hindgut | Body Mass | Gut Mass |
| --- | --- | --- | --- | --- | --- | --- | --- | --- | --- |
| 3rd | WT-Conv | 0.59 | 0.0091* | 0.0106* | 0.0170* | 0.0014* | 0.8361 | -- | -- |
|  | Conv-GF |  | 0.0382* | 0.8005 | 0.3746 | 1.0000 | 0.0846 |  |  |
|  | WT-GF |  | 0.7480 | 0.0303* | 0.1717 | 0.0001* | 0.0109* |  |  |
| 4th | WT-Conv | 0.17 | 0.57 | 0.1676 | 0.08 | 0.3055 | 0.0042* | 0.0806 | 0.0659 |
|  | Conv-GF |  |  |  |  |  | 0.6586 |  |  |
|  | WT-GF |  |  |  |  |  | 0.0000* |  |  |
| 5th | WT-Conv | 0.36 | 0.1935 | 0.0028* | 0.24 | 0.5281 | 0.0022* | 0.4398 | 0.7717 |
|  | Conv-GF |  | 0.0058* | 0.0244* |  |  | 0.0002* |  |  |
|  | WT-GF |  | 0.0000* | 0.0054* |  |  | 0.0000* |  |  |
| 6th | WT-Conv^1^ | 0.1093 | 0.2947 | 0.4389 | 0.1264 | 0.3686 | 0.0191* | 0.0363* | 0.0727 |

Table 1.
